# Cross-Cultural Adaptation and Psychometric Evaluation of the Italian Version of the Systemic Lupus Erythematosus Symptom Checklist

**DOI:** 10.3390/healthcare14213572

**Published:** 2026-10-22

**Authors:** Sofia Magrin, Chiara Lioce, Massimo Radin, Irene Cecchi, Alice Barinotti, Lois Ira Capulong, Fabio Petrelli, Giovanni Cangelosi, Savino Sciascia, Alessio Conti

**Affiliations:** 1 Department of Clinical and Biological Sciences, University of Torino, 10043 Orbassano, Italy; 2 Department of Public Health and Pediatrics, University of Torino, 10124 Torino, Italy; 3 University Center of Excellence on Nephrologic, Rheumatologic and Rare Diseases (ERK-Net, ERN-Reconnect and RITA-ERN Member), with Nephrology and Dialysis Unit and Center of Immuno-Rheumatology and Rare Diseases (CMID), Coordinating Center of the Interregional Network for Rare Diseases of Piedmont and Aosta Valley, San Giovanni Bosco Hub Hospital, ASL Città di Torino, 10154 Torino, Italyalice.barinotti@unito.it (A.B.);; 4 School of Pharmacy, Experimental Medicine and “Stefania Scuri” Public Health Department, University of Camerino, 62032 Camerino, Italy; 5 Research and Innovation Unit for Health Professions, San Luigi Gonzaga University Hospital, 10043 Orbassano, Italy

**Keywords:** systemic lupus erythematosus, patient-reported outcome measures, symptom burden, psychometric evaluation, factor analysis, quality of life

## Abstract

**Background/Objectives**: Systemic lupus erythematosus (SLE) causes a wide, fluctuating range of disease- and treatment-related symptoms that objective indices capture only partially. The SLE Symptom Checklist (SSC) is a disease-specific patient-reported measure of symptom occurrence and perceived burden, but no Italian version exists, and its latent structure has never been examined with confirmatory methods. This study aimed to translate, culturally adapt and psychometrically evaluate the Italian SSC in patients with SLE, and to characterize its dimensionality. **Methods**: A cross-sectional study was conducted in 331 patients (301 with SLE, with or without antiphospholipid syndrome [APS]; 30 with primary APS as an exploratory subgroup). Items were recoded to a single 0–4 ordinal metric (0 = absent; 1–4 = burden). Analyses included exploratory factor analysis of polychoric correlations, within-sample ordinal confirmatory factor analysis, cross-validation, bifactor modeling and a sensitivity analysis excluding primary APS. Concurrent validity was assessed against the EQ-5D visual analog scale (EQ-VAS), GAD-7 and PHQ-9. **Results**: An exploratory 23-item short form, derived with loading-based retention rules, comprised five symptom clusters (pain/somatic–systemic, neuropsychological/mood, skin/treatment-related, appetite/weight, photosensitivity) and showed acceptable within-sample fit (CFI = 0.96; RMSEA = 0.055), but limited discriminant validity. Bifactor analysis showed a dominant general factor (ECV = 0.61; omega hierarchical = 0.85). The 38-item total score showed high internal consistency (α = 0.92) and correlated with EQ-VAS (ρ = −0.49), GAD-7 (ρ = 0.41) and PHQ-9 (ρ = 0.57); results were similar after excluding primary APS. **Conclusions**: The Italian SSC showed good internal consistency, content validity and concurrent validity, and should be considered an essentially unidimensional measure of symptom burden with five descriptive symptom clusters requiring independent confirmation. Its usefulness for longitudinal monitoring remains to be established.

## 1. Introduction

Systemic Lupus Erythematosus (SLE) and Antiphospholipid Syndrome (APS) are chronic autoimmune diseases with complex, multifactorial pathogenesis, marked clinical heterogeneity, and an unpredictable course. The interplay of genetic predisposition with environmental, hormonal, and immunological factors drives the production of specific autoantibodies, leading to a broad spectrum of immune-mediated manifestations that affect multiple organs and systems, including the skin, kidneys, musculoskeletal, cardiovascular, and nervous systems [1]. APS may occur as a primary condition or, more frequently, in association with SLE, and is characterized by recurrent arterial and/or venous thromboses and obstetric complications [2,3]. The relapsing-remitting course of these diseases contributes to a fluctuating symptom burden that affects daily functioning, self-management, and quality of life (QoL), with considerable variability both across individuals and within the same individual over time [4]. SLE predominantly affects women of childbearing age, with incidence rates nearly six times higher than in men. Globally, the disease has an estimated incidence of 5.14 cases per 100,000 person-years and a prevalence of 43.7 cases per 100,000 individuals, corresponding to approximately 3.4 million affected people worldwide [5]. APS is a rare disease, with reported incidence rates ranging from 0.75 to 2.7 cases per 100,000 person-years and prevalence estimates between 6 and 50 cases per 100,000 individuals [6]. The marked heterogeneity of SLE and APS also poses important diagnostic and management challenges. Current classification systems, including the 2012 SLICC and 2019 EULAR/ACR criteria for SLE and the 2023 ACR/EULAR criteria for APS, combine clinical manifestations with immunological findings to support disease classification [7,8,9]. Because several classification criteria rely on the recognition and interpretation of patient-reported manifestations, accurate symptom assessment is fundamental not only for disease classification but also for monitoring disease evolution, identifying disease flares, supporting clinical decision-making, and optimizing healthcare resource utilization. From a clinical and nursing perspective, however, symptom assessment remains challenging. The subjective and fluctuating nature of symptoms is often only partially captured by physician-reported disease activity indices, which may not adequately reflect the patient’s experience [10,11,12]. Patient-Reported Outcome Measures (PROMs) have therefore become increasingly relevant for integrating patients’ perspectives into routine care, supporting person-centered assessment, and complementing traditional clinical evaluation. Nevertheless, reliable disease-specific instruments capable of comprehensively assessing both symptom occurrence and its perceived burden remain limited. Several PROMs are currently available for patients with SLE, including generic health-status measures such as the Medical Outcomes Study Short Form-36 (MOS-SF-36), disease-specific instruments including the Lupus Quality of Life questionnaire (LupusQoL), the Systemic Lupus Erythematosus-specific Quality of Life questionnaire (SLEQoL), and the Systemic Lupus Activity Questionnaire (SLAQ), as well as symptom-specific measures such as the Fatigue Severity Scale (FSS) and the Functional Assessment of Chronic Illness Therapy-Fatigue scale (FACIT-Fatigue) [13,14,15,16,17,18,19]. However, generic instruments may be insufficiently sensitive to disease-specific issues, whereas symptom-specific questionnaires evaluate only selected manifestations. Likewise, disease-specific QoL instruments primarily assess the impact of disease on functioning and well-being rather than providing a comprehensive evaluation of individual symptoms and their perceived burden.

To address this need, Grootscholten et al. (2003) developed the SLE Symptom Checklist (SSC), a disease-specific PROM comprising 38 symptoms commonly experienced by patients with SLE [20]. The SSC combines symptom occurrence with patients’ ratings of symptom-related burden, thereby providing a more comprehensive assessment of symptom burden than instruments focusing exclusively on health status or QoL. Validation studies conducted in Dutch and English populations demonstrated satisfactory psychometric properties, including good internal consistency, test–retest reliability, construct validity, and responsiveness to treatment-induced changes. Owing to its focus on symptom burden, the SSC represents a potentially valuable instrument for routine clinical assessment, longitudinal symptom monitoring, evaluation of treatment outcomes, and research. More recently, Japanese, Portuguese, and Chinese adaptations have also confirmed its validity in patients with SLE [21,22,23]. Despite these encouraging findings, important methodological limitations remain. Across previous validation studies, the SSC has generally been used as a 38-item unidimensional checklist, while psychometric evaluation has mainly focused on internal consistency, test–retest reliability, and concurrent validity. Its latent structure has never been investigated using confirmatory factor analysis, and the original exploratory factor analysis, conducted on a relatively small sample, failed to support the hypothesized multidimensional structure. Consequently, whether a more parsimonious multidimensional model better represents the construct measured by the SSC remains unclear [24,25]. To date, no validated Italian version of the SSC is available, limiting both clinical practice and research by preventing the use of a culturally adapted instrument specifically designed to assess the symptom burden experienced by patients with SLE. Furthermore, given the substantial clinical overlap between SLE and APS, exploring the applicability of the SSC in patients with SLE and APS may provide preliminary evidence supporting a broader patient-centered assessment of symptom burden across systemic autoimmune diseases [26]. Therefore, the primary aim of this study was to translate and culturally adapt the SSC into Italian and to evaluate its psychometric properties in patients with SLE, while examining its underlying dimensional structure and reliability. As a secondary exploratory objective, we also evaluated the applicability of the instrument in a subgroup of patients with APS included in the study sample.

## 2. Materials and Methods

The cross-cultural adaptation and psychometric evaluation of the Italian SSC followed Sousa et al. [27] and were reported according to Kottner et al. [28] (Supporting File S1) and the COSMIN reporting guideline (Supporting File S2) [29] through the following phases.

### 2.1. Translation and Cross-Cultural Adaptation

#### 2.1.1. Phase I: Forward Translation

The SSC and its accompanying instructions were translated from English into Italian by two independent bilingual translators, both of whom are native Italian speakers. One translator possessed expertise in the clinical domain, while the other specialized in education. The translation preserved a conversational tone to maintain the conceptual integrity of the original document, employing a literal translation that integrated expressions deemed appropriate from both medical and cultural perspectives.

#### 2.1.2. Phase II: Synthesis

Both SSC translations were submitted to the research team, which subsequently assembled, summarized, cross-verified, and reviewed the final version of the scale. Discrepancies were addressed through discussion, resulting in a mutually agreed adaptation. No items from the original scale were excluded.

#### 2.1.3. Phase III: Backward Translation

The final version of the SSC was translated from Italian into English by a certified translator whose native language was English. This translation was subsequently provided to one of the original authors of the scale, who verified the conceptual equivalence of the Italian version despite minor vocabulary discrepancies and authorized its use. The process was concluded upon the acquisition of a definitive Italian version.

#### 2.1.4. Phase IV: Expert Committee

The Italian SSC was evaluated by a panel comprising 8 healthcare professionals (including three rheumatologists, three clinical pathologists (included for their role in the laboratory diagnosis and monitoring of SLE and APS), a nephrologist, and a registered nurse) from Italian hospitals not involved in data collection, and 12 individuals diagnosed with SLE and/or APS selected among representatives of a patient association, to determine its content validity. Panel members had no relationship with the patients included in the main study and did not take part in it. Content validity pertains to the degree to which the instrument’s items accurately and comprehensively represent the symptom burden that characterizes SLE and APS. Panel members independently rated each item on a 5-point Likert scale, ranging from 1 (not representative) to 5 (strongly representative). The Italian SSC achieved a content validity index of 0.91 (I-CVI computed among all 20 panel members as the proportion rating the item 4 or 5; scale-level CVI as the mean I-CVI), with all items exceeding the established validity cutoff of 0.78, thereby supporting its content validity [30].

#### 2.1.5. Phase V: Preliminary Testing

A preliminary study was undertaken to assess the face validity of the Italian SSC. Face validity refers to the extent to which the instrument’s wording is sufficiently clear to effectively describe the symptom burden experienced by individuals with SLE and/or APS. A sample of 13 participants diagnosed with SLE and/or APS who met the inclusion criteria was recruited and asked to complete the Italian SSC to assess the appropriateness of the items from a face-validity perspective. The items were rated using a ten-point Likert scale, ranging from 0 (not appropriate) to 10 (highly appropriate). The Italian SSC achieved a face validity score of 9.75 out of 10. Data from this cohort were not incorporated into the main study.

### 2.2. Procedure

Participants were enrolled during follow-up appointments at the outpatient clinics of the University Center of Excellence on Nephrologic, Rheumatologic and Rare Diseases (ERK-Net, ERN-Reconnect and RITA-ERN Member), with the Nephrology and Dialysis Unit and the Center of Immuno-Rheumatology and Rare Diseases (CMID) of the San Giovanni Bosco Hospital, Turin, Italy. Adults (≥18 years) with a confirmed diagnosis of SLE and/or APS were included consecutively from December 2025 to May 2026. Administration of the entire set of instruments took 20 to 30 min and was conducted in a separate area of the waiting room to ensure participant privacy. The following data were collected: Sociodemographic data were collected using a purpose-designed questionnaire. Information was gathered on participants’ age, gender, educational level, marital status, number of children, and employment status. Clinical information on SLE and/or APS was self-reported and included diagnosis and comorbidities.

The presence and burden of symptoms were evaluated using the 38-item Italian translation (Supplementary Material, Table S1) of the SSC [20]. To assess test–retest reliability and evaluate the temporal stability of the questionnaire, the first 20 participants were asked to complete the questionnaire a second time, 48 h after the initial administration. The scale was completed online through a secure platform (LimeSurvey). This time interval was deemed appropriate to minimize the likelihood of changes in participants’ clinical condition and to reduce the risk of dropout between administrations. The quality of life was evaluated utilizing the EQ-5D visual analog scale (EQ-VAS). Developed by the EuroQol Group, this instrument is extensively used to measure overall quality of life [31], including among people with autoimmune disorders [32]. The EQ-VAS facilitates patients’ self-assessment of their health status on a vertical visual analog scale, with endpoints designated as 0 (worst possible health) and 100 (best achievable health). Depression was assessed with the Patient Health Questionnaire-9 (PHQ-9), a versatile instrument designed to screen for depression severity and suicidal ideation [33]. The questionnaire comprises nine items that incorporate diagnostic criteria for depression alongside symptoms of depressive episodes. Scores on the PHQ-9 range from 0 to 27, categorizing depression severity into mild, moderate, moderately severe, and severe levels. The PHQ-9 has been used to screen for depression in pediatric [34] and adult [35] lupus populations. Anxiety was measured using the Generalized Anxiety Disorder-7 (GAD-7). This seven-item self-administered questionnaire yields scores from 0 to 21, identifying mild, moderate, and severe anxiety [36]. It has been rated as moderately good for screening panic, social anxiety, and post-traumatic stress disorders and has been used in people with SLE [35].

### 2.3. Data Analysis

#### 2.3.1. Descriptive Analysis

The results of the administered instruments, together with participants’ sociodemographic and clinical characteristics, were summarized using descriptive statistics (means, standard deviations, and frequencies).

#### 2.3.2. Structural Validity

The dimensionality of the Italian SSC was initially explored through exploratory factor analysis (EFA) on the polychoric correlation matrix using minimum-residual extraction and oblique (oblimin) rotation, as symptom dimensions were expected to be correlated. Items were administered in the original two-step format (symptom present in the past month: no/yes; if yes, burden: not, a little, quite or extremely burdensome) and recoded for analysis into a single 0–4 ordinal variable (0 = absent; 1–4 = increasing burden), which reproduces the original scoring [20]. Since several items showed pronounced floor effects, all factor analyses were based on polychoric rather than Pearson correlations [37]. In all confirmatory models, the items were declared ordinal and estimated with diagonally weighted least squares on the polychoric correlation matrix, with robust standard errors and a mean- and variance-adjusted test statistic (WLSMV) [38]; scaled fit indices are reported. With 331 participants, the five-factor CFA estimated 125 free parameters (23 loadings, 10 factor correlations and 92 thresholds), i.e., 2.6 cases per free parameter overall and 10.0 per loading or factor correlation. The number of retained factors was primarily determined through parallel analysis, scree plot inspection, and the interpretability of competing solutions. Item retention followed pre-specified criteria: items were retained when they demonstrated a primary standardized loading ≥ 0.40 and no secondary loading ≥ 0.30 on another factor; items failing either criterion were removed [39]. These criteria were applied at the EFA stage only. Three post hoc, content-based deviations were made (Table S2): item 29 (cross-loading 0.31) was retained to preserve the photosensitivity pair; item 16 (decreased appetite), which loaded negatively, was excluded because reverse-scoring a symptom within a burden score is clinically meaningless; and item 30 (fits/seizures), which met the criteria, was excluded as clinically incongruent with its factor. Sampling adequacy was evaluated using the Kaiser–Meyer–Olkin (KMO) statistic and Bartlett’s test of sphericity. A KMO value ≥ 0.80 and a significant Bartlett’s test (*p* < 0.05) were considered indicative of adequate factorability [40]. The factor structure identified through EFA was subsequently examined using confirmatory factor analysis (CFA). Model fit was evaluated using the comparative fit index (CFI), Tucker–Lewis index (TLI), and root mean square error of approximation (RMSEA), adopting conventional criteria for acceptable fit (CFI and TLI ≥ 0.95; RMSEA ≤ 0.06). Because EFA and CFA were performed on the same sample, the CFA was interpreted as an assessment of the internal coherence of the proposed solution rather than an independent validation. Accordingly, in this study, the term validation refers to cross-cultural adaptation, content validity, reliability and concurrent validity, whereas the factor structure is regarded as provisional pending confirmation in an independent sample. Stability of the standardized factor loadings was examined through non-parametric bootstrapping, reporting bootstrap means and 95% percentile confidence intervals. As an internal stability check, repeated five-fold cross-validation (20 × 5 folds) was performed: in each fold, the five-factor and unidimensional models for the same 23 items were estimated in the training partition, and the model-implied polychoric correlation matrix was compared with the polychoric matrix of the held-out partition using the maximum-likelihood discrepancy function (lower values indicating better reproduction). Because item selection and the number of factors were fixed beforehand, this procedure is not an independent test of model selection. To examine the relative contribution of a general symptom burden factor and the specific symptom dimensions, a bifactor model was estimated including one general factor loading on all retained items and orthogonal specific factors. The two-item specific factors were identified by constraining their loadings to equality. Explained common variance (ECV), proportion of uncontaminated correlations (PUC), omega hierarchical, and omega hierarchical subscale coefficients were calculated to evaluate the extent to which the SSC could be interpreted as an essentially unidimensional measure. A unidimensional model of all 38 items and a Schmid–Leiman decomposition of the 38-item polychoric matrix were used to evaluate the structure of the full-length total score. A sensitivity analysis repeated the EFA, CFA, and reliability analyses in the SLE-spectrum subsample (primary APS excluded) and, more conservatively, after also excluding the patients who reported other associated conditions.

#### 2.3.3. Construct Validity

Discriminant validity of the retained factors was evaluated using the Fornell–Larcker criterion by comparing the square root of the average variance extracted (AVE) for each factor with its correlations with the remaining factors. Discriminant validity was considered adequate when the square root of the AVE exceeded the corresponding inter-factor correlations. Composite reliability (CR) and AVE were calculated from the standardized CFA loadings, considering AVE ≥ 0.50 as evidence for convergent validity and CR ≥ 0.70 as acceptable. Concurrent validity was examined by correlating the SSC total score and factor scores with the EQ-VAS, GAD-7, and PHQ-9. Correlations of the total scores were hypothesized to be negative with the EQ-VAS and positive with the GAD-7 and PHQ-9, and at least moderate in magnitude (|ρ| ≥ 0.30), given the conceptual overlap between symptom burden, perceived health and psychological distress [41]. Concurrent-validity analyses used pairwise-complete data (EQ-VAS *n* = 313, four EQ-VAS entries recorded as free text were treated as missing; GAD-7 *n* = 269; PHQ-9 *n* = 268); no imputation was performed. Given the ordinal nature of the data and non-normal score distributions, Spearman’s rank correlation coefficients were calculated.

#### 2.3.4. Reliability

Reliability was evaluated for both the total SSC score and the dimensions identified through factor analysis. Internal consistency was assessed using Cronbach’s α and, where appropriate, the Spearman–Brown coefficient; values ≥ 0.70 were considered satisfactory [41]. Test–retest reliability was evaluated using the intraclass correlation coefficient (ICC), two-way mixed, absolute agreement, single measures, quantifying the proportion of total score variability attributable to differences between patients across repeated administrations. Measurement error was expressed as the standard error of measurement (SEM = SD × √(1 − ICC)) and the smallest detectable change (SDC = 1.96 × √2 × SEM) [41]. Floor and ceiling effects were considered present if >15% of respondents obtained the minimum or maximum total score.

#### 2.3.5. Statistical Software

Descriptive statistics and test–retest ICCs were computed in IBM SPSS Statistics version 29 (IBM Corp., Armonk, NY, USA). All other analyses were performed in R version 4.3.3 (R Foundation for Statistical Computing, Vienna, Austria): polychoric correlations, parallel analysis, exploratory factor analysis (minimum-residual extraction, oblimin rotation), Schmid–Leiman decomposition, Cronbach’s α and Spearman correlations with the psych (version 2.4.1) and GPArotation (version 2024.2-1) packages; confirmatory, bifactor and cross-validation models with lavaan (version 0.6-17). Bootstrap resampling and bifactor indices (ECV, PUC, omega coefficients) were computed from the lavaan solutions with custom code. Statistical significance was set at *p* < 0.05.

## 3. Results

### 3.1. Sample Description

Of the 354 eligible patients consecutively approached, 18 declined to participate and 5 were excluded because of incomplete data; the final sample comprised 331 participants (93.5%), the majority of whom were diagnosed with SLE (*n* = 301; 90.9%). Of these, 68 were diagnosed with both SLE and APS, while the remaining 30 participants (9.1%) had APS exclusively. Thirteen patients classified as having SLE also reported other conditions (e.g., Sjögren’s syndrome, neuropsychiatric lupus) in a free-text field. The mean age of the cohort was 46.1 years (SD ± 13.3). Male participants (*n* = 19) represented 5.7% of the sample. Additionally, 17.8% of participants (*n* = 59) reported emergency department visits within the previous year (Table 1).

Most participants (*n* = 276; 83.4%) reported experiencing ten or more symptoms (Table 2). The most frequently reported symptoms included fatigue (*n* = 285; 86.1%), joint pain (*n* = 266; 80.4%), muscle weakness (*n* = 248; 74.9%), mood changes (*n* = 237; 71.6%), and loss of concentration (*n* = 229; 69.2%). Conversely, the least commonly reported symptoms were genital sores (*n* = 26; 7.9%), pimples/acne (*n* = 42; 12.7%), and fits/seizures (*n* = 44; 13.3%). The total SSC score (38 items; range 0–152) differed across diagnostic groups (Kruskal–Wallis *p* = 0.001), being highest in patients with SLE and APS (mean 58.0 ± 26.1), intermediate in those with SLE alone (49.4 ± 27.2), and lowest in those with primary APS (36.3 ± 22.6). No floor or ceiling effects were observed (38-item total: 0.6% and 0% of respondents at the minimum and maximum score; 23-item total: 1.5% and 0%).

Participants reported a mean EQ-VAS score of 63.7 (SD ± 19.9). Almost half of the sample (*n* = 130; 48.3%) showed moderate to severe anxiety. Regarding PHQ-9 scores, 48.1% of participants (*n* = 129) reported moderate to severe depressive symptoms (Table 3).

### 3.2. Structural Validity

The sampling adequacy of the Italian SSC was deemed excellent, with a KMO measure of 0.897. Bartlett’s test of sphericity yielded a significant result (χ^2^ = 4133.8, *p* < 0.001), computed on Pearson item correlations and supporting factorability. Parallel analysis of the polychoric correlation matrix supported the extraction of five factors. The first eigenvalue (12.8) was much larger than the second (2.3), indicating the presence of a predominant common dimension alongside subordinate factors. Application of retention rules yielded an interpretable solution comprising 23 items distributed across five dimensions (Table 4): pain/somatic–systemic (factor 1), neuropsychological/mood (factor 2), skin/treatment-related (factor 3), appetite/weight (factor 4), and photosensitivity (factor 5). Of the 23 items, 22 met the retention rule; item 29 was retained, and items 16 and 30 were excluded as post hoc content-based deviations. A complete description of the item identification and exclusion process is provided in Table S2.

Within the derivation sample, the 23-item five-factor model showed acceptable fit (scaled χ^2^(220) = 443.3, *p* < 0.001; CFI = 0.959; TLI = 0.953; RMSEA = 0.055, 90% CI 0.048–0.063; SRMR = 0.069). Standardized loadings ranged from 0.48 (item 18, ‘facial hair growth’) to 0.88 (item 38, ‘weight gain’), and all bootstrap 95% confidence intervals excluded zero (Table 5).

**Figure 1:**
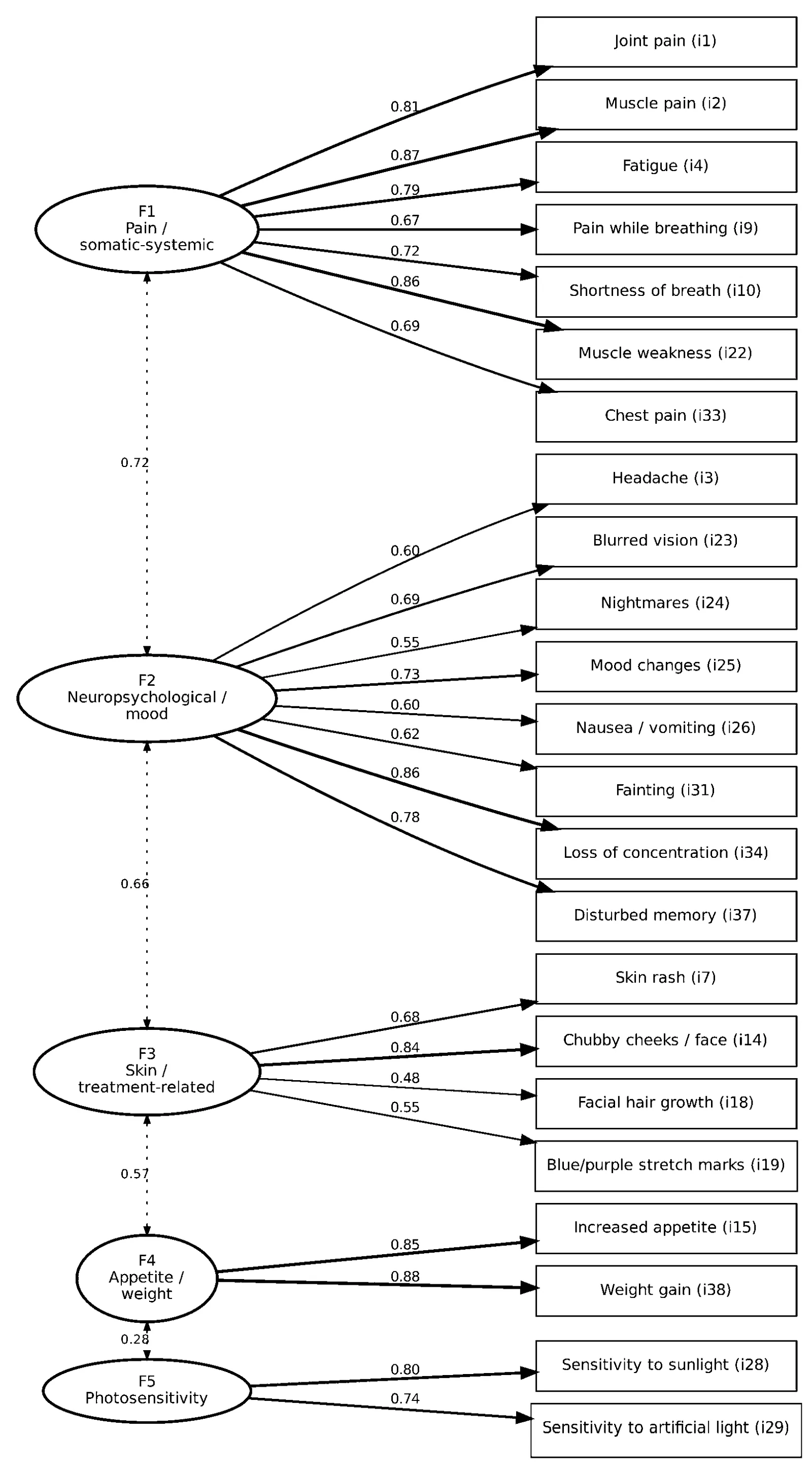
Confirmatory factor structure of the Italian SSC (23-item short form; items declared ordinal, WLSMV estimation). Single-headed arrows show standardized loadings; dotted double-headed arrows show correlations between adjacent factors (the full latent correlation matrix is reported in Table S4).

The five factors were strongly intercorrelated (Figure 1). Notably, the pain/somatic–systemic and neuropsychological/mood factors demonstrated a high correlation coefficient (r = 0.72), indicating a potential shared, overarching dimension of overall symptom burden. This shared variance was examined through model comparison and bifactor modeling (Supplementary Material, Tables S3 and S4). In cross-validation, the five-factor model reproduced held-out correlation matrices slightly better than a unidimensional model of the same items (mean discrepancy 40.8 vs. 42.3; lower in 99 of 100 folds), a within-sample stability check only. AVE was below 0.50 for two factors (neuropsychological/mood, 0.47; skin/treatment-related, 0.42), and the Fornell–Larcker criterion was not met for the F1–F2 and F2–F3 pairs. AVE was below 0.50 for four of the five factors, and the Fornell–Larcker criterion was not met for several factor pairs. A bifactor model revealed the general factor explained most variance (ECV = 0.61) and dominated scores (omega hierarchical = 0.85; omega total = 0.95), meaning that about 85% of the variance of the 23-item total score reflects a single general dimension. After removing the general factor, the specific factors held little reliable variance (omega-hierarchical-subscale: F1 = 0.28, F2 = 0.15, F3 = 0.39, F5 = 0.37), with appetite/weight change (F4 = 0.57) retaining a substantial unique contribution. With a PUC of 0.77, these indices suggest the Italian SSC is essentially unidimensional for scoring purposes, and the five factors reflect thematic symptom clusters rather than independent subscales. For the full 38-item checklist, a unidimensional model did not reach conventional fit criteria (CFI = 0.887; TLI = 0.880; RMSEA = 0.061), whereas a Schmid–Leiman decomposition indicated a dominant general factor (ωH = 0.73; ωt = 0.96) (Table S3). In sensitivity analyses restricted to the SLE spectrum (*n* = 301 and *n* = 288), the short-form items kept their factor assignment (23/23 and 22/23), and model fit, bifactor indices and reliability were essentially unchanged; however, when the retention rules were re-applied de novo, the two photosensitivity items fell below the loading threshold (Tables S5 and S6).

### 3.3. Reliability

The 23-item total scale showed good internal consistency (Cronbach’s α = 0.89), and the corrected item–total correlations were all positive (range 0.27–0.64), with no item substantially lowering the overall coefficient (Cronbach’s α-if-item-deleted range 0.88–0.89), indicating that all items contributed consistently to the total score. At the factor level, internal consistency was good for the two larger dimensions, the 7-item pain/somatic–systemic (Cronbach’s α = 0.84) and 8-item neuropsychological/mood (Cronbach’s α = 0.80), and below the 0.70 threshold for the 4-item skin/treatment-related factor (Cronbach’s α = 0.62). For the two-item factors (appetite/weight and photosensitivity), the Spearman–Brown coefficient was adequate for appetite/weight change (*r* = 0.74) and below the 0.70 threshold for photosensitivity (*r* = 0.62). The full 38-item Italian SSC total score showed high internal consistency (Cronbach’s α = 0.92; 95% CI 0.90–0.93). In the test–retest subsample (*n* = 20; 48 h interval), the ICC was 0.90 (95% CI 0.75–0.96) for the 38-item total and 0.92 (95% CI 0.76–0.97) for the 23-item total. Similarly, the symptom clusters showed a good test–retest reliability; respectively, ICC for pain/somatic–systemic was 0.91 (CI = 0.75–0.96), 0.90 (CI = 0.73–0.95) for neuropsychological/mood, 0.89 (CI = 0.71–0.94) for skin/treatment-related (factor 3), 0.88 (CI = 0.69–0.95) for appetite/weight, and 0.89 (CI = 0.72–0.96) for photosensitivity. The SEM was 8.6 points for the 38-item total (SD = 27.1; ICC = 0.90) and 5.2 points for the 23-item total (SD = 18.4; ICC = 0.92), corresponding to SDCs of 23.7 and 14.4 points, respectively; a minimal clinically important difference could not be estimated in this cross-sectional design.

### 3.4. Concurrent Validity

Overall, the Italian SSC correlated with established measures of QoL and psychological distress, consistent with its intended construct (Table 6). All correlations were statistically significant (*p* < 0.01) and in the expected directions, and the total-score correlations met the hypothesized magnitude, supporting the instrument’s concurrent validity. The 38-item total score was negatively associated with EQ-VAS (ρ = −0.49) and positively associated with anxiety (GAD-7, ρ = 0.41) and depression (PHQ-9, ρ = 0.57), with similar values for the 23-item short form (ρ = −0.49, 0.43 and 0.59), indicating that a higher symptom burden corresponded to poorer QoL and greater psychological distress. The pattern across the specific-factor scores was theoretically consistent. The pain/somatic–systemic factor showed the strongest association with impaired QoL (EQ-VAS, ρ = −0.54), whereas the neuropsychological/mood factor was most strongly related to the psychological measures (PHQ-9, ρ = 0.59; GAD-7, ρ = 0.47). The skin/treatment-related (ρ = −0.24 to 0.33), appetite/weight (ρ = −0.23 to 0.35), and photosensitivity (ρ = −0.22 to 0.27) factors showed weaker but consistently significant correlations, in line with their more circumscribed content.

## 4. Discussion

This study cross-culturally adapted the SSC into Italian and evaluated its psychometric properties, extending previous validation studies [20,21,22,23] through advanced psychometric analyses appropriate for ordinal data. The instrument showed high internal consistency, preliminary evidence of short-term test–retest reliability, content validity, and expected associations with quality of life, anxiety and depression. Although a 23-item, five-factor structure was identified, bifactor modeling supported interpretation of the SSC total score as the primary measure of overall symptom burden, while the five dimensions represent clinically meaningful symptom clusters rather than independent subscales. This result should not be regarded as a shortcoming of the instrument but as a precise characterization of what it measures, and it reconciles the present findings with the original conception of the scale. Indeed, Grootscholten et al. designed the SSC as a unidimensional checklist and reported that their exploratory analysis did not recover the conceptually anticipated dimensions [20]. Likewise, the recent Chinese adaptation, using exploratory analysis only, retained 33 items and identified a markedly different ten-factor solution [23], suggesting that the SSC substructure does not replicate across languages, even though the total score’s internal consistency has remained high. Analyzed here using methods suited to ordinal indicators and extended to a bifactor framework, our data both confirm essential unidimensionality and offer a plausible explanation: the symptom dimensions are real and clinically interpretable, but they largely reflect a common burden that the total score captures efficiently.

The emergence of a skin/treatment-related cluster, encompassing skin rash, chubby cheeks and face, facial hair growth, and blue or purple stretch marks, along with a distinct photosensitivity dimension, is conceptually plausible. We hypothesize that the former partly reflects the well-documented cutaneous and metabolic effects of corticosteroid therapy [42]. However, skin rash is primarily a disease manifestation, and the association with treatment was not tested in this study, while photosensitivity is a characteristic cutaneous feature of lupus, listed in the 1997 ACR criteria but not as a standalone item in the 2012 SLICC or 2019 EULAR/ACR criteria [7,8]. Their emergence herein, where they were not identified in the original study, may be attributable to the larger sample size, the ordinal modeling of symptom burden, or genuine differences in the perception and reporting of these symptoms [43]. Notably, appetite and weight changes constituted the only dimension functioning as a partially independent subscale, exhibiting substantial unique variance and relatively weak external correlations; this might reflect treatment-related metabolic effects [44], a hypothesis that requires testing against treatment data. The pattern of concurrent validity supported the clinical interpretability within the clinical framework. Overall symptom burden demonstrated consistent associations with poorer QoL [45], as well as elevated levels of anxiety and depression [46]. These associations adhered to theoretically anticipated patterns: the neuropsychological/mood dimension exhibited the strongest correlation with depression and anxiety, while the pain/somatic–systemic dimension was most strongly linked to reduced QoL. This differentiation, observed even within an essentially unidimensional instrument, suggests that, within the 23-item short form, the thematic clusters may provide HCPs with a descriptive profile of the specific domain where a patient’s burden is most concentrated [47]. Generic and disease-specific QoL instruments emphasize the impact of illness on functioning and well-being [48], whereas the SSC focuses on the occurrence and perceived burden of individual symptoms. The two approaches are complementary, and a symptom-based measure can be particularly well suited to capturing treatment-related and cutaneous manifestations that QoL instruments tend to under-represent. The most frequently reported symptoms in our sample, fatigue, joint pain, and muscle weakness, are consistent with the wider SLE literature, in which fatigue is repeatedly identified as the most prevalent and burdensome symptom [49]. The present work extends the cross-cultural evidence for the SSC beyond the original Dutch/English version [20] and the subsequent Japanese, Portuguese, and Chinese adaptations [21,22,23]. To our knowledge, it is the first to characterize the latent structure of the Italian SSC using confirmatory and bifactor methods, providing a preliminary basis for its scoring and interpretation.

### 4.1. Perspectives for Practice

The Italian SSC showed good internal consistency, content validity and concurrent validity for the systematic assessment of symptom burden in patients with SLE. Its essentially unidimensional structure supports the use of the total score as an overall indicator of symptom burden, while the identified symptom clusters may provide additional clinical insight into the predominant areas of patient-reported distress. In clinical practice, the SSC may complement physician-based assessments by capturing aspects of the illness experience that are not fully reflected by traditional disease activity measures [20,50]. Its integration into routine care could support person-centered assessment and may help identify patients who could benefit from further evaluation; however, as responsiveness and predictive value were not assessed, these uses remain hypotheses requiring longitudinal confirmation. Furthermore, the availability of a culturally adapted Italian version provides a useful instrument for clinical research and international comparisons of patient-reported outcomes in systemic autoimmune diseases.

### 4.2. Strengths and Limitations

This study presents several limitations. Primarily, the factor structure was both derived via EFA and subsequently tested through CFA within the same sample. Consequently, the confirmatory fit indices obtained under these conditions may be partly circular and tend to be overly optimistic. Thus, the fit indices should be interpreted as indicative of internal coherence rather than evidence of replicability. Bootstrapping and repeated cross-validation were used only as internal stability checks: item selection and the number of factors were fixed before cross-validation, and the unidimensional comparator was fitted to items selected for the five-factor solution. These procedures do not replace the need for confirmation of the factor structure in an independent sample, which constitutes the essential next step. Furthermore, two of the five factors (appetite/weight and photosensitivity) were defined by only two items, making them just-identified and less stable; their reliability, estimated with the Spearman–Brown coefficient from a single inter-item correlation, is less precise and not directly comparable with the α values of the longer subscales. The bifactor model was also highly parameterized relative to the sample size; given the unbalanced factor sizes, these indices should be interpreted cautiously. The bifactor indices should therefore be regarded as approximate, and the photosensitivity factor, in particular, should be retained partly on clinical grounds despite a marginal cross-loading and requires confirmation. Third, since the factors share a large common variance, convergent and discriminant validity were limited, and subscale scores should not be used as independent measures. Fifteen of the original 38 items, including rare or treatment-dependent symptoms, were not retained, creating a more parsimonious scale but excluding some clinically relevant, infrequent manifestations. Because the checklist is partly formative, removing items was not meant to discard clinical content. The 23-item, five-factor solution combines rule-based selection with three post hoc content-based decisions and was not fully reproduced when the rules were re-applied in the SLE-spectrum subsample; it is therefore an exploratory short form; the full 38-item SSC remains necessary for comprehensive symptom coverage. HCPs wanting full coverage can administer all 38 items and use the 38-item total score; descriptive profiling by symptom cluster applies only to the 23 items of the short form, as the remaining 15 items have no cluster assignment. A fourth limitation is that, although the sample mainly comprised SLE patients, it also included patients with APS. The instrument was designed for SLE, and applying it to the APS subgroup was exploratory; although excluding primary APS left the structure essentially unchanged, the symptom profile of primary APS may differ from that of SLE, so the factor structure should not be assumed to transfer unchanged; measurement invariance across diagnostic subgroups could not be tested given their limited size. Larger samples are needed for subgroup validation. Finally, the inclusion of participants from a single tertiary center, combined with the cross-sectional design of our study, may limit generalizability. Test–retest reliability was estimated in a small subsample over a 48 h interval, which may have favored recall, and with a change from in-clinic to online administration; moreover, sensitivity to change was not assessed and should be evaluated in longitudinal studies, ideally alongside HCP-rated disease activity and disease-activity indexes to further clarify the instrument’s position relative to objective measures. In fact, the absence of a clinician-rated anchor derived from an assessment of disease activity (e.g., SLEDAI-2K), damage, or a physician’s general assessment represents a key limitation of this study and will need to be addressed in future validity analyses. Nevertheless, our study has several strengths: a formal, multi-step cross-cultural adaptation process with documented content and face validity, a reasonably large sample for a rare disease, complete item-level data, the use of polychoric correlations and an estimator appropriate for ordinal responses, and a comprehensive analytic strategy combining EFA and CFA with model comparison, internal cross-validation, discriminant-validity testing and a bifactor decomposition. Lastly, with 331 participants, the sample exceeded the commonly recommended minimum of 300 participants for factor analysis, with approximately 14 participants per retained item [51].

## 5. Conclusions

The Italian SSC showed good internal consistency, content validity and concurrent validity for assessing symptom burden in individuals with SLE. Within-sample CFA and bifactor modeling indicate that it should be interpreted as an essentially unidimensional instrument: the total score reflects overall symptom burden, while five symptom clusters, identified in an exploratory 23-item short form, provide a descriptive profile. By providing a first internal evaluation of the instrument’s underlying structure, this study provides preliminary support for the use of the Italian SSC in both research and clinical settings. In clinical practice, the SSC may facilitate systematic assessment of patient-reported symptoms, complement traditional disease evaluations, and support person-centered care. Confirmation of the factor structure in an independent sample remains a priority for future research.

## Figures and Tables

**Table 1:** Sample characteristics (*n* = 331).

Variables	*n*	%	Mean (SD)
Age (years)			46.1 (±13.3)
Females	312	94.3	
SLE	233	70.4	
APS	30	9.1	
SLE and APS	68	20.5	
High school or University grade	256	77.3	
Presence of children	177	53.5	
Unemployed	61	18.5	

Legend. SD: standard deviation; *n*: number; %: percentage; SLE: Systemic Lupus Erythematosus; APS: Antiphospholipid Syndrome. SLE includes 13 patients who also reported other conditions.

**Table 2:** Frequencies and burden severity of the reported symptoms (*n* = 331).

Symptom	*n*	%	Quite or Extreme Burden n (%)
Fatigue	285	86.1	225 (68.0)
Joint pain	266	80.4	189 (57.1)
Muscle weakness	248	74.9	198 (59.8)
Mood changes	237	71.6	183 (55.3)
Loss of concentration	229	69.2	184 (55.6)
Muscle pain	225	68.0	166 (50.2)
Sensitivity to sunlight	220	66.5	171 (51.7)
Spontaneous bruises	207	62.5	99 (29.9)
Headache	203	61.3	139 (42.0)
Red/painful eyes	196	59.2	131 (39.6)
Disturbed memory	195	58.9	145 (43.8)
Shortness of breath	187	56.5	125 (37.8)
Skin rash	179	54.1	112 (33.8)
Itch	178	53.8	113 (34.1)
Stomach complaints	178	53.8	135 (40.8)
Blurred vision	176	53.2	134 (40.5)
Muscle cramps	165	49.8	105 (31.7)
Vulnerable skin	159	48.0	108 (32.6)
Weight gain	152	45.9	129 (39.0)
Sensitivity to artificial light	146	44.1	103 (31.1)
‘White’ fingers in cold weather	137	41.4	75 (22.7)
Hair loss	135	40.8	96 (29.0)
Ankle edema	135	40.8	86 (26.0)
Poor wound healing	134	40.5	72 (21.8)
Ulcers in mouth/throat	127	38.4	66 (19.9)
Increased appetite	122	36.9	94 (28.4)
Nausea/vomiting	117	35.3	70 (21.1)
Nightmares	103	31.1	70 (21.1)
Chubby cheeks/face	95	28.7	77 (23.3)
Chest pain	93	28.1	60 (18.1)
Fainting	76	23.0	52 (15.7)
Decreased appetite	73	22.1	30 (9.1)
Facial hair growth	71	21.5	54 (16.3)
Blue/purple stretch marks	71	21.5	36 (10.9)
Pain while breathing	55	16.6	42 (12.7)
Fits/seizures	44	13.3	30 (9.1)
Pimples/acne	42	12.7	26 (7.9)
Genital sores	26	7.9	20 (6.0)

Legend. *n*: number; %: percentage. Percentages are calculated over the whole cohort (*n* = 331), i.e., the prevalence of that burden level in the full sample.

**Table 3:** Scores obtained in the administered questionnaires.

Instrument (Cut-Off)	Mean (SD)	*n*	%
EQ-VAS	63.7 (±19.9)	313	100
GAD-7	10.1 (±5.2)	269	100
Minimal anxiety (0–4)		36	13.4
Mild anxiety (5–9)		103	38.3
Moderate anxiety (10–14)		67	24.9
Severe anxiety (15–21)		63	23.4
PHQ-9	10.3 (±5.4)	268	100
Minimal or no depression (0–4)		35	13.1
Mild depression (5–9)		104	38.8
Moderate depression (10–14)		78	29.1
Moderately severe depression (15–19)		33	12.3
Severe depression (20–27)		18	6.7

Legend. SD: standard deviation; EQ-VAS: EQ-5D visual analog scale; GAD-7: Generalized Anxiety Disorder-7; PHQ-9: Patient Health Questionnaire-9; *n*: number; %: percentage. Four EQ-VAS entries recorded as free text were treated as missing.

**Table 4:** Exploratory factor loadings for the Italian SSC.

	Symptom	F1	F2	F3	F4	F5	h^2^	Factor
1	Joint pain	0.63	–	–	–	–	0.58	F1
2	Muscle pain	0.65	–	–	–	–	0.67	F1
3	Headache	–	0.61	–	–	–	0.42	F2
4	Fatigue	0.60	–	–	–	–	0.59	F1
5	Ulcers in mouth/throat	0.30	–	–	–	–	0.28	–
6	Hair loss	–	–	0.38	–	–	0.34	–
7	Skin rash	–	–	0.43	–	–	0.44	F3
8	Red/painful eyes	–	0.39	–	–	0.43	0.53	–
9	Pain while breathing	0.88	–	–	–	–	0.65	F1
10	Shortness of breath	0.66	–	–	–	–	0.53	F1
11	‘White’ fingers in cold weather	–	–	0.34	–	–	0.27	–
12	Itch	–	–	–	–	–	0.22	–
13	Ankle edema	0.31	–	0.34	–	–	0.36	–
14	Chubby cheeks/face	–	–	0.44	–	–	0.53	F3
15	Increased appetite	–	–	–	0.77	–	0.75	F4
16	Decreased appetite	–	–	–	−0.66	–	0.54	– ^a^
17	Pimples/acne	–	0.43	–	–	−0.45	0.43	–
18	Facial hair growth	–	–	0.77	–	–	0.57	F3
19	Blue/purple stretch marks	–	–	0.73	–	–	0.54	F3
20	Spontaneous bruises	–	–	0.40 ^b^	–	–	0.43	–
21	Poor wound healing	–	–	–	–	–	0.34	–
22	Muscle weakness	0.54	–	–	–	–	0.63	F1
23	Blurred vision	–	0.54	–	–	–	0.51	F2
24	Nightmares	–	0.59	–	–	–	0.36	F2
25	Mood changes	–	0.46	–	–	–	0.50	F2
26	Nausea/vomiting	–	0.48	–	–	–	0.45	F2
27	Stomach complaints	–	0.33	–	–	–	0.39	–
28	Sensitivity to sunlight	–	–	–	–	0.46	0.47	F5
29	Sensitivity to artificial light	–	0.31	–	–	0.41	0.40	F5 ^a^
30	Fits/seizures	–	–	0.42	–	–	0.30	– ^a^
31	Fainting	–	0.54	–	–	–	0.44	F2
32	Genital sores	–	0.40 ^b^	–	–	–	0.19	–
33	Chest pain	0.66	–	–	–	–	0.51	F1
34	Loss of concentration	–	0.79	–	–	–	0.70	F2
35	Muscle cramps	0.38	–	–	–	–	0.43	–
36	Vulnerable skin	–	–	0.46	–	0.39	0.52	–
37	Disturbed memory	–	0.76	–	–	–	0.57	F2
38	Weight gain	–	–	–	0.70	–	0.71	F4
	Variance explained (%)	10.9	10.7	7.4	4.9	4.5		

N.B. Pattern loadings ≥ |0.30| are shown (polychoric correlations; minimum-residual extraction; oblimin rotation; *n* = 331). h^2^ = communality (diagonal of ΛΦΛ′). ^a^ Post hoc, content-based deviations from the retention rule (see Table S2). ^b^ 0.396 and 0.399 before rounding (below threshold). ‘Skin/treatment-related’ is an interpretative label; its association with treatment exposure was not tested. Lines in grey color refer to retained items.

**Table 5:** Standardized CFA loadings of the Italian SSC.

Factor	Item	Std. Loading	95% CI
F1—Pain/somatic–systemic	Joint pain (i1)	0.81	0.74–0.87
	Muscle pain (i2)	0.87	0.83–0.92
	Fatigue (i4)	0.79	0.72–0.84
	Pain while breathing (i9)	0.67	0.54–0.81
	Shortness of breath (i10)	0.72	0.64–0.78
	Muscle weakness (i22)	0.86	0.81–0.90
	Chest pain (i33)	0.69	0.57–0.80
F2—Neuropsychological/mood	Headache (i3)	0.60	0.50–0.71
	Blurred vision (i23)	0.69	0.59–0.77
	Nightmares (i24)	0.55	0.43–0.67
	Mood changes (i25)	0.74	0.65–0.81
	Nausea/vomiting (i26)	0.60	0.49–0.72
	Fainting (i31)	0.62	0.50–0.73
	Loss of concentration (i34)	0.86	0.81–0.91
	Disturbed memory (i37)	0.78	0.69–0.85
F3—Skin/treatment-related	Skin rash (i7)	0.68	0.58–0.79
	Chubby cheeks/face (i14)	0.84	0.72–0.93
	Facial hair growth (i18)	0.48	0.29–0.64
	Blue/purple stretch marks (i19)	0.55	0.37–0.70
F4—Appetite/weight	Increased appetite (i15)	0.85	0.74–0.96
	Weight gain (i38)	0.88	0.77–0.98
F5—Photosensitivity	Sensitivity to sunlight (i28)	0.80	0.69–0.91
	Sensitivity to artificial light (i29)	0.74	0.61–0.84

N.B. Items declared ordinal; WLSMV estimation. 95% CI = percentile bootstrap interval (500 resamples, 482 admissible solutions). Model fit: scaled χ^2^(220) = 443.3; CFI = 0.959; TLI = 0.953; RMSEA = 0.055 (90% CI 0.048–0.063); SRMR = 0.069. ‘Skin/treatment-related’ is an interpretative label; its association with treatment exposure was not tested.

**Table 6:** Correlation between Italian SSC and other administered questionnaires.

SSC Score	EQ-VAS (*n* = 313)	GAD-7 (*n* = 269)	PHQ-9 (*n* = 268)
Total SSC score (38-item)	−0.49 **	0.41 **	0.57 **
Total SSC score (23-item short form)	−0.49 **	0.43 **	0.59 **
Factor 1—Pain/somatic–systemic	−0.54 **	0.30 **	0.44 **
Factor 2—Neuropsychological/mood	−0.37 **	0.47 **	0.59 **
Factor 3—Skin/treatment-related	−0.24 **	0.25 **	0.33 **
Factor 4—Appetite/weight change	−0.23 **	0.20 **	0.35 **
Factor 5—Photosensitivity	−0.22 **	0.17 **	0.27 **

Legend. Spearman’s ρ. EQ-VAS: EuroQol visual analog scale; GAD-7: Generalized Anxiety Disorder-7; PHQ-9: Patient Health Questionnaire-9. ** *p* < 0.01 (two-tailed). ‘Skin/treatment-related’ is an interpretative label; its association with treatment exposure was not tested.

## Data Availability

The data that support the findings of this study are available from the corresponding authors upon reasonable request, due to privacy and ethical considerations. The R code used for the analyses is available from the corresponding author upon reasonable request.

## Supplementary Materials

The following supporting information can be downloaded at: https://www.mdpi.com/article/10.3390/healthcare14213572/s1, File S1. GRRAS checklist—Guidelines for Reporting Reliability and Agreement Studies; File S2. COSMIN reporting checklist; Table S1. Italian SSC items (wording administered) and corresponding original English items; Table S2. Item-level exploratory factor analysis of all 38 items; Table S3. Comparison of competing measurement models; Table S4. Convergent and discriminant validity of the five factors: average variance extracted (AVE), composite reliability (CR) and Fornell–Larcker matrix; Table S5. Sensitivity analyses: full sample versus SLE-spectrum subsamples; Table S6. Item-level exploratory factor analysis re-estimated de novo in the SLE-spectrum subsample.

## Abbreviations

The following abbreviations are used in this manuscript:

ACR	American College of Rheumatology
APS	antiphospholipid syndrome
AVE	average variance extracted
CFA	confirmatory factor analysis
CFI	comparative fit index
CI	confidence interval
COSMIN	Consensus-based Standards for the selection of health Measurement Instruments
CR	composite reliability
DWLS	diagonally weighted least squares
ECV	explained common variance
EFA	exploratory factor analysis
EQ-5D	EuroQol five-dimension questionnaire
EQ-VAS	EuroQol visual analog scale
EULAR	European League Against Rheumatism
FACIT-Fatigue	Functional Assessment of Chronic Illness Therapy–Fatigue
FSS	Fatigue Severity Scale
GAD-7	Generalized Anxiety Disorder 7-item scale
HCP	healthcare professional
ICC	intraclass correlation coefficient
KMO	Kaiser–Meyer–Olkin
LupusQoL	Lupus Quality of Life questionnaire
MOS-SF-36	Medical Outcomes Study 36-Item Short Form Health Survey
PHQ-9	Patient Health Questionnaire-9
PROM	patient-reported outcome measure
PUC	proportion of uncontaminated correlations
QoL	quality of life
RMSEA	root mean square error of approximation
SD	standard deviation
SDC	smallest detectable change
SEM	standard error of measurement
SLAQ	Systemic Lupus Activity Questionnaire
SLE	systemic lupus erythematosus
SLEQoL	Systemic Lupus Erythematosus-specific Quality of Life questionnaire
SLICC	Systemic Lupus International Collaborating Clinics
SPSS	Statistical Package for the Social Sciences
SRMR	standardized root mean square residual
SSC	Systemic Lupus Erythematosus Symptom Checklist
TLI	Tucker–Lewis index
WLSMV	Weighted least squares, mean- and variance-adjusted
