# Supplementary material for: Cross-Cultural Adaptation and Psychometric Evaluation of the Italian Version of the Systemic Lupus Erythematosus Symptom Checklist
Source: Healthcare (Basel). 2026 Oct 22;14(21):3572. doi: 10.3390/healthcare14213572 (PMC13762971; doi:10.3390/healthcare14213572)
Supplement: Supplementary file 1 [file healthcare-14-03572-s001.zip › healthcare-4585991-supplementary.pdf]

Supplementary File S1. GRRAS checklist – Guidelines for Reporting Reliability and Agreement Studies

Kottner, J.; Audigé, L.; Brorson, S.; et al. Guidelines for Reporting Reliability and Agreement Studies (GRRAS) were proposed. J. Clin. Epidemiol. 2011, 64, 96–106.

Section	No.	Checklist item	Reported in (section – page)
TITLE AND ABSTRACT	1	Identify in title or abstract that interrater/intrarater reliability or agreement was investigated.	Title ("Psychometric Evaluation"); Abstract (reliability: internal consistency) – p. 1
INTRODUCTION	2	Name and describe the diagnostic or measurement device of interest explicitly.	Section 1, second paragraph – pp. 2–3
	3	Specify the subject population of interest.	Section 1 – pp. 2–3
	4	Specify the rater population of interest (if applicable).	Not applicable (self-administered patient-reported measure; no raters involved)
	5	Describe what is already known about reliability and agreement and provide a rationale for the study (if applicable).	Section 1, second paragraph – pp. 2–3
METHODS	6	Explain how the sample size was chosen. State the determined number of raters, subjects/objects, and replicate observations.	Section 2.2 (test–retest: first 20 participants, two administrations) – p. 4; Section 2.3.2 (sample size and cases per parameter) – p. 5; Section 4.2 – p. 16
	7	Describe the sampling method.	Section 2.2 (consecutive enrolment) – p. 4; Section 3.1 – p. 7
	8	Describe the measurement/rating process (e.g., time interval between repeated measurements, availability of clinical information, blinding).	Section 2.2 (48-hour interval; retest online via LimeSurvey) – p. 4
	9	State whether measurements/ratings were conducted independently.	Section 2.2 (independent self-completion at each administration) – p. 4
	10	Describe the statistical analysis.	Section 2.3.4 (ICC two-way mixed, absolute agreement, single measures; SEM; SDC) – p. 7; Section 2.3.5 – p. 7
RESULTS	11	State the actual number of raters and subjects/objects which were included and the number of replicate observations which were conducted.	Section 3.1 – p. 7; Section 3.3 (n = 20; two administrations) – p. 13
	12	Describe the sample characteristics of raters and subjects (e.g., training, experience).	Section 3.1 and Table 1 – p. 7
	13	Report estimates of reliability and agreement including measures of statistical uncertainty.	Section 3.3 (ICC with 95% CI; SEM; SDC) – p. 13
DISCUSSION	14	Discuss the practical relevance of results.	Sections 4.1 and 4.2 – pp. 15–16
AUXILIARY MATERIAL	15	Provide detailed results if possible (e.g., online).	Supplementary Material, Tables S1–S6

Page numbers refer to the revised clean manuscript.

Supplementary File S2. COSMIN reporting checklist

Gagnier, J.J.; Lai, J.; Mokkink, L.B.; Terwee, C.B. COSMIN reporting guideline for studies on measurement properties of patient-reported outcome measures. Qual. Life Res. 2021, 30, 2197–2218. Item wording is abbreviated; see the original guideline for the full text.

Section / property	Item	Content (abbreviated)	Reported in (section – page)
TITLE	T1	Name and version of the PROM	Title – p. 1
	T2	Measurement properties studied	Title (“Psychometric Evaluation”); Abstract – p. 1
	T3	Study sample / population	Abstract (patients with SLE; primary APS as exploratory subgroup) – p. 1
ABSTRACT	A1–A7	PROM, properties, design, sample, methods, main results with precision, conclusions	Abstract – p. 1
INTRODUCTION	I1	PROM name, development and structure	Section 1, second paragraph – pp. 2–3
	I2	Target population	Section 1 – p. 2
	I3	Original development and previous studies cited	Section 1, second paragraph [20–23] – pp. 2–3
	I4	Current knowledge and rationale	Section 1, second paragraph – pp. 2–3
	I5	Definitions of specialized terms	Sections 2.1.4, 2.1.5 and 2.3.2 – pp. 4–6
	I6	Objectives and hypotheses	Section 1, last paragraph – p. 3; hypotheses in Section 2.3.3 – p. 6
METHODS – general	GM1	Study design	Section 2.2 (cross-sectional; test–retest subsample) – p. 4
	GM2	Participant selection and eligibility	Section 2.2 – p. 4; Section 3.1 – p. 7
	GM3	How and when the PROM was administered	Section 2.2 – p. 4; Section 2.3.2 (response format) – p. 5; Table S1
	GM4	Other data collected; follow-up time points	Section 2.2 (EQ-VAS, GAD-7, PHQ-9; retest at 48 h) – p. 4
	GM5	Sample size	Section 2.3.2 (cases per free parameter) – p. 5; Section 4.2 – p. 16
	GM6	Statistical methods, criteria and software	Sections 2.3.1–2.3.5 – pp. 5–7
	GM7	Handling of missing data	Section 2.3.3 (pairwise-complete data; no imputation) – p. 6; Section 3.1 – p. 7
	GM8	Post hoc analyses and rationale	Section 2.3.2 (post hoc item-retention deviations; sensitivity analyses) – pp. 5–6; Table S2
RESULTS – general	GR1	Amount of and reasons for missing data	Section 3.1 – p. 7; Tables 3 and 6 – pp. 9, 13
	GR2	Participant characteristics, PROM scores	Section 3.1; Tables 1–3 – pp. 7–9
	GR3	Sample size per analysis	Tables 3 and 6 – pp. 9, 13; Table S5
DISCUSSION	D1	Comparison with criteria for good measurement properties	Sections 4 and 4.2 – pp. 14–16
	D2	Practical relevance	Section 4.1 – p. 15
	D3	Strengths and limitations	Section 4.2 – pp. 15–16
	D4	Generalizability	Section 4.2 – p. 16
	D5	Need for modification of the PROM	Section 4.2 (exploratory short form; full 38 items for coverage) – p. 16
	D6	Future research	Sections 4.2 and 5 – p. 16
CONCLUSIONS	C1	Overall conclusions per measurement property	Section 5 – p. 16
OTHER	O1	Conflicts of interest and funding	Funding and Conflicts of Interest statements – p. 17
Content validity	CV1	Rating of item relevance by patients/professionals	Section 2.1.4 – p. 4
	CV2	Assessment of comprehensiveness	Section 2.1.4 (expert committee) – p. 4
	CV3	Assessment of comprehensibility	Section 2.1.5 (preliminary testing) – p. 4

Section / property	Item	Content (abbreviated)	Reported in (section – page)
	CV4–CV7	Results on relevance, response options/recall period, comprehensiveness, comprehensibility	Sections 2.1.4–2.1.5 – p. 4; Table S1
Structural validity	SV1	Factor analysis methods and results	Sections 2.3.2 and 3.2 – pp. 5–6, 9–12; Tables 4–5; Figure 1; Tables S2–S6
	SV2	IRT/Rasch	Not applicable
Internal consistency	IC1–IC2	Per (essentially) unidimensional scale; α/ω	Sections 2.3.4 and 3.3 – pp. 7, 13; bifactor indices in Section 3.2 – pp. 11–12; Table S3
	IC3	Dichotomous items	Not applicable
Cross-cultural validity / measurement invariance	CCV1–CCV3	Comparison of groups; multigroup CFA/DIF	Not assessed (subgroups too small; Section 4.2 – p. 16)
Reliability	R1	Number of measurements, administration, interval	Section 2.2 (n = 20; 48 h; online retest) – p. 4
	R2	Statistical method (ICC model)	Section 2.3.4 (two-way mixed, absolute agreement, single measures) – p. 7
	R3	Methods to improve reliability	Not applicable
Measurement error	ME1–ME2	Measurements; SEM, SDC	Section 2.3.4 – p. 7; Section 3.3 – p. 13
Criterion validity	CriV1–CriV3	Gold standard	Not applicable (no gold standard)
Hypotheses testing for construct validity	ConV1	Comparator instruments and their properties	Section 2.2 – p. 4
	ConV2	Comparison groups	Not applicable (known-groups validity not assessed; diagnostic subgroups described only, Section 3.1 – p. 8)
	ConV3	Hypotheses on direction and magnitude	Section 2.3.3 – p. 6
	ConV4	Statistical methods	Section 2.3.3 (Spearman's ϱ) – p. 6
	ConV5	Results in relation to hypotheses	Section 3.4; Table 6 – p. 13
Responsiveness	Resp1–Resp9	Change scores, anchors	Not applicable (cross-sectional design; Section 4.2 – p. 16)

Page numbers refer to the revised clean manuscript.

Supplementary Materials

Table S1. Italian SSC items (wording administered) and corresponding original English items.

No.	Italian item	Original English item
1	Dolore alle articolazioni	Joint pain
2	Dolore ai muscoli	Muscle pain
3	Mal di testa	Headache
4	Fatica o senso di affaticamento anche in seguito ad attività leggere	Fatigue
5	Afte/Ulcere in bocca o in gola	Ulcers in mouth/throat
6	Perdita di capelli o peli/ Alopecia	Hair loss
7	Arrossamento della pelle	Skin rash
8	Secchezza, arrossamento, dolore agli occhi	Red/painful eyes
9	Dolore durante la respirazione	Pain while breathing
10	Mancanza di fiato o senso di fiato corto	Shortness of breath
11	Dita bianche durante la stagione fredda	'White' fingers in cold weather
12	Prurito	Itch
13	Edema/Gonfiore delle caviglie	Ankle oedema
14	Gonfiore del viso e/o delle guance	Chubby cheeks/face
15	Aumento dell'appetito	Increased appetite
16	Riduzione dell'appetito	Decreased appetite
17	Presenza o aumento di acne	Pimples/acne
18	Crescita anomala di peluria sul viso	Facial hair growth
19	Presenza di smagliature o striature blu/viola sulla pelle	Blue/purple stretch marks
20	Comparsa di lividi spontanei	Spontaneous bruises
21	Ridotta guarigione delle ferite	Poor wound healing
22	Debolezza muscolare	Muscle weakness
23	Vista sfocata	Blurred vision
24	Incubi notturni	Nightmares
25	Sbalzi di umore	Mood changes
26	Nausea/Vomito	Nausea/vomiting
27	Bruciore/Disturbi di stomaco	Stomach complaints
28	Sensibilità alla luce del sole	Sensitivity to sunlight
29	Sensibilità alle luci artificiali	Sensitivity to artificial light
30	Convulsioni o movimenti muscolari involontari	Fits
31	Svenimento o senso di venir meno	Fainting
32	Comparsa di piaghe genitali	Genital sores
33	Dolore al torace	Chest pain
34	Mancanza di concentrazione o mente offuscata	Loss of concentration
35	Crampi muscolari	Muscle cramps
36	Aumentata fragilità della pelle	Vulnerable skin
37	Disturbi di memoria	Disturbed memory
38	Aumento di peso	Weight gain

Note. Instructions and response format as administered (original two-step format): «Nell'ultimo mese ha avuto…?» No (0) / Sì → «Se sì, quanto le ha dato fastidio?» Per niente (1) / Un po' (2) / Abbastanza (3) / Moltissimo (4). [AUTHORS: verify against the administered questionnaire and replace if the wording differs.] For analysis, the two steps were combined into a single 0–4 ordinal score (0 = symptom absent; 1 = present, not burdensome; 2 = a little; 3 = quite; 4 = extremely burdensome), which reproduces the original scoring. Total score range: 0–152 (38 items) and 0–92 (23-item short form). In the main-text tables item 30 is labelled "Fits/seizures", item 11 "'White' fingers in cold weather" and item 17 "Pimples/acne".

Table S2. Item-level exploratory factor analysis of all 38 items.

Item	F1	F2	F3	F4	F5	h^2	Retained	Reason / criterion
1. Joint pain	0.63	–	–	–	–	0.58	Yes (F1)	Primary loading ≥ 0.40, no cross-loading ≥ 0.30
2. Muscle pain	0.65	–	–	–	–	0.67	Yes (F1)	Primary loading ≥ 0.40, no cross-loading ≥ 0.30
3. Headache	–	0.61	–	–	–	0.42	Yes (F2)	Primary loading ≥ 0.40, no cross-loading ≥ 0.30
4. Fatigue	0.60	–	–	–	–	0.59	Yes (F1)	Primary loading ≥ 0.40, no cross-loading ≥ 0.30
5. Ulcers in mouth/throat	0.30	–	–	–	–	0.28	No	Primary loading < 0.40 (0.30)
6. Hair loss	–	–	0.38	–	–	0.34	No	Primary loading < 0.40 (0.38)
7. Skin rash	–	–	0.43	–	–	0.44	Yes (F3)	Primary loading ≥ 0.40, no cross-loading ≥ 0.30
8. Red/painful eyes	–	0.39	–	–	0.43	0.53	No	Cross-loading ≥ 0.30 (0.39 on F2)
9. Pain while breathing	0.88	–	–	–	–	0.65	Yes (F1)	Primary loading ≥ 0.40, no cross-loading ≥ 0.30
10. Shortness of breath	0.66	–	–	–	–	0.53	Yes (F1)	Primary loading ≥ 0.40, no cross-loading ≥ 0.30
11. 'White' fingers in cold weather	–	–	0.34	–	–	0.27	No	Primary loading < 0.40 (0.34)
12. Itch	–	–	–	–	–	0.22	No	Primary loading < 0.40 (0.29)
13. Ankle oedema	0.31	–	0.34	–	–	0.36	No	Primary loading < 0.40 (0.34) and cross-loading ≥ 0.30 (0.31 on F1)
14. Chubby cheeks/face	–	–	0.44	–	–	0.53	Yes (F3)	Primary loading ≥ 0.40, no cross-loading ≥ 0.30
15. Increased appetite	–	–	–	0.77	–	0.75	Yes (F4)	Primary loading ≥ 0.40, no cross-loading ≥ 0.30
16. Decreased appetite	–	–	–	−0.66	–	0.54	No	Met the rule (−0.66 on F4) but excluded post hoc[a]: negative loading; reverse-scoring a symptom within a burden score is clinically meaningless
17. Pimples/acne	–	0.43	–	–	−0.45	0.43	No	Cross-loading ≥ 0.30 (0.44 on F2; −0.45 on F5)
18. Facial hair growth	–	–	0.77	–	–	0.57	Yes (F3)	Primary loading ≥ 0.40, no cross-loading ≥ 0.30
19. Blue/purple stretch marks	–	–	0.73	–	–	0.54	Yes (F3)	Primary loading ≥ 0.40, no cross-loading ≥ 0.30
20. Spontaneous bruises	–	–	0.40[b]	–	–	0.43	No	Primary loading < 0.40 (0.396)
21. Poor wound healing	–	–	–	–	–	0.34	No	Primary loading < 0.40 (0.29)
22. Muscle weakness	0.54	–	–	–	–	0.63	Yes (F1)	Primary loading ≥ 0.40, no cross-loading ≥ 0.30
23. Blurred vision	–	0.54	–	–	–	0.51	Yes (F2)	Primary loading ≥ 0.40, no cross-loading ≥ 0.30
24. Nightmares	–	0.59	–	–	–	0.36	Yes (F2)	Primary loading ≥ 0.40, no cross-loading ≥ 0.30
25. Mood changes	–	0.46	–	–	–	0.50	Yes (F2)	Primary loading ≥ 0.40, no cross-loading ≥ 0.30
26. Nausea/vomiting	–	0.48	–	–	–	0.45	Yes (F2)	Primary loading ≥ 0.40, no cross-loading ≥ 0.30
27. Stomach complaints	–	0.33	–	–	–	0.39	No	Primary loading < 0.40 (0.33)
28. Sensitivity to sunlight	–	–	–	–	0.46	0.47	Yes (F5)	Primary loading ≥ 0.40, no cross-loading ≥ 0.30
29. Sensitivity to artificial light	–	0.31	–	–	0.41	0.40	Yes (F5)	Post hoc deviation[a]: retained with item 28 as photosensitivity pair despite cross-loading 0.31 on F2
30. Fits/seizures	–	–	0.42	–	–	0.30	No	Met the rule (0.42 on F3) but excluded post hoc[a]: content (fits/seizures) clinically incongruent with the skin/treatment-related factor
31. Fainting	–	0.54	–	–	–	0.44	Yes (F2)	Primary loading ≥ 0.40, no cross-loading ≥ 0.30
32. Genital sores	–	0.40[b]	–	–	–	0.19	No	Primary loading < 0.40 (0.399)
33. Chest pain	0.66	–	–	–	–	0.51	Yes (F1)	Primary loading ≥ 0.40, no cross-loading ≥ 0.30
34. Loss of concentration	–	0.79	–	–	–	0.70	Yes (F2)	Primary loading ≥ 0.40, no cross-loading ≥ 0.30
35. Muscle cramps	0.38	–	–	–	–	0.43	No	Primary loading < 0.40 (0.38)
36. Vulnerable skin	–	–	0.46	–	0.39	0.52	No	Cross-loading ≥ 0.30 (0.39 on F5)
37. Disturbed memory	–	0.76	–	–	–	0.57	Yes (F2)	Primary loading ≥ 0.40, no cross-loading ≥ 0.30
38. Weight gain	–	–	–	0.70	–	0.71	Yes (F4)	Primary loading ≥ 0.40, no cross-loading ≥ 0.30

Note. Same output as Table 4 of the main text. Loadings ≥ |0.30| shown; – = loading < |0.30|. h^2 = communality, computed as the diagonal of $\Lambda\Phi\Lambda'$ (oblique solution). Retention rule (applied at the EFA stage only; no loading criterion was applied at the CFA stage): primary loading ≥ 0.40 and no secondary loading ≥ 0.30. [a]Post hoc, content-based deviations from the rule (items 16, 29 and 30). [b]Values before rounding 0.396 (item 20) and 0.399 (item 32), i.e., below the 0.40 threshold. In the ordinal CFA all 23 retained items loaded ≥ 0.40 (lowest: item 18, 0.48); item 30, if added to F3, would load 0.55 with unchanged fit (CFI 0.959; RMSEA 0.053). Variance explained by F1–F5 (sum of squared pattern loadings/38): 10.9%, 10.7%, 7.4%, 4.9%, 4.5%. Leading eigenvalues: 12.77, 2.32, 2.04.

Table S3. Comparison of competing measurement models .

Model	Items	Free par. (thresholds)	Scaled χ^2 (df)	CFI	TLI	RMSEA (90% CI)	SRMR	CV-F
Correlated five-factor	23	125 (92)	443.3 (220)	0.959	0.953	0.055 (0.048–0.063)	0.069	40.8
Unidimensional	23	115 (92)	913.0 (230)	0.874	0.861	0.095 (0.088–0.101)	0.097	42.3
Bifactor, 1 general + 5 specific	23	136 (92)	386.2 (209)	0.967	0.960	0.051 (0.043–0.059)	0.065	–
Unidimensional (all items)	38	189 (151)	1478.5 (665)	0.887	0.880	0.061 (0.057–0.065)	0.092	–

Note. Scaled (mean- and variance-adjusted) fit statistics. Free parameters include item thresholds; excluding thresholds, the five-factor model estimated 33 parameters (23 loadings and 10 factor correlations), i.e., 2.6 cases per free parameter overall and 10.0 per loading/factor correlation. CV-F = mean maximum-likelihood discrepancy between the model-implied polychoric correlation matrix estimated in the training partition and the polychoric correlation matrix of the held-out partition, over repeated five-fold cross-validation (20 × 5 folds; lower = better reproduction); the five-factor model had the lower value in 99 of 100 folds. CV-F is used here only as a distance between correlation matrices (not as an estimator) and is comparable only between models fitted to the same items. Item selection and the number of factors were fixed before cross-validation and were not re-estimated within folds: the procedure is an internal stability check, not an independent test of model selection, and the unidimensional comparator was fitted to items selected for the five-factor solution. AIC/BIC are not reported because they are not defined under WLSMV estimation. Bifactor identification: specific factors orthogonal to each other and to the general factor; loadings of the two two-item specific factors constrained equal; solution admissible. Bifactor indices: ECV = 0.61; ωt = 0.95; ωH = 0.85; PUC = 0.77; ωHS F1 = 0.28, F2 = 0.15, F3 = 0.39, F4 = 0.57, F5 = 0.37. For the 38-item total, a Schmid–Leiman decomposition (five group factors) gave ωt = 0.96 and ωH = 0.73.

Table S4. Convergent and discriminant validity of the five factors: average variance extracted (AVE), composite reliability (CR) and Fornell–Larcker matrix.

Factor	AVE	CR	F1	F2	F3	F4	F5
F1 Pain/somatic-systemic	0.60	0.91	**0.78**				
F2 Neuropsychological/mood	0.47	0.88	0.72	**0.69**			
F3 Skin/treatment-related	0.42	0.74	0.61	0.66	**0.65**		
F4 Appetite/weight	0.75	0.86	0.49	0.48	0.57	**0.86**	
F5 Photosensitivity	0.60	0.75	0.61	0.63	0.55	0.28	**0.77**

Note. Values from the ordinal CFA (Table 5). Diagonal (bold) = √AVE; below diagonal = latent inter-factor correlations. AVE < 0.50 for F2 and F3 indicates limited convergent validity; the Fornell–Larcker criterion is not met where an inter-factor correlation exceeds the √AVE of either factor (F1–F2: 0.72 > 0.69; F2–F3: 0.66 > 0.65). The substantial shared variance among factors is examined with the bifactor model (main text, Section 3.2; Table S3).

Table S5. Sensitivity analyses: full sample versus SLE-spectrum subsamples.

Indicator	Full sample	SLE spectrum (primary APS excluded)	SLE spectrum, also excluding other self-reported diagnoses
N	331	301	288
First / second eigenvalue (polychoric, 38 items)	12.77 / 2.32	12.71 / 2.36	12.76 / 2.26
Tucker congruence with full-sample EFA factors (range)	1.00	0.95–0.99	0.88–0.98
Of the 23 short-form items, same primary factor in the re-estimated EFA	23	23	22[a]
Items meeting the retention rule de novo (of 38)	24[b]	26[c]	24[d]
Of the 23 short-form items, meeting the rule de novo	22	21	19
CFA, 23-item five-factor model: scaled χ^2 (df)	443.3 (220)	417.7 (220)	396.3 (220)
CFI / TLI	0.959 / 0.953	0.960 / 0.954	0.962 / 0.956
RMSEA / SRMR	0.055 / 0.069	0.055 / 0.071	0.053 / 0.070
Range of standardized CFA loadings	0.48–0.88	0.45–0.89	0.44–0.90
Bifactor ECV / ωt / ωH (23 items)	0.61 / 0.95 / 0.85	0.63 / 0.95 / 0.87	0.64 / 0.95 / 0.87
Cronbach's α, 23-item / 38-item total	0.89 / 0.92	0.89 / 0.92	0.89 / 0.92
ϱ 23-item total with EQ-VAS / GAD-7 / PHQ-9	−0.49 / 0.43 / 0.59	−0.48 / 0.39 / 0.57	−0.49 / 0.41 / 0.56
ϱ 38-item total with EQ-VAS / GAD-7 / PHQ-9	−0.49 / 0.41 / 0.57	−0.48 / 0.38 / 0.55	−0.49 / 0.40 / 0.55
n for EQ-VAS / GAD-7 / PHQ-9	313 / 269 / 268	285 / 241 / 240	275 / 231 / 230

Note. Diagnostic group as reported in Table 1: SLE alone (n = 233, including 13 patients who reported additional diagnoses in a free-text field), SLE with associated APS (n = 68), primary APS (n = 30). In each subsample the polychoric matrix, five-factor EFA (all 38 items, with factors matched to the full-sample solution by Tucker congruence), retention rule, CFA, bifactor model and reliability were re-estimated de novo; item-level results for n = 301 are given in Table S6. [a]Item 29 (sensitivity to artificial light) shifted to F2. [b]All short-form items except item 29, plus items 16 and 30. [c]All short-form items except items 28 and 29, plus items 20, 30, 32, 35 and 36. [d]All short-form items except items 25, 26, 28 and 29, plus items 16, 20, 30, 32 and 36. All correlations p < 0.001.

Table S6. Item-level exploratory factor analysis re-estimated de novo in the SLE-spectrum subsample.

Item	F1	F2	F3	F4	F5	h²	Meets rule (n = 301)	Meets rule (n = 288)	Short form (n = 331)
1. Joint pain	0.81	–	–	–	–	0.66	Yes (F1)	Yes (F1)	F1
2. Muscle pain	0.75	–	–	–	–	0.70	Yes (F1)	Yes (F1)	F1
3. Headache	–	0.58	–	–	–	0.42	Yes (F2)	Yes (F2)	F2
4. Fatigue	0.68	–	–	–	–	0.61	Yes (F1)	Yes (F1)	F1
5. Ulcers in mouth/throat	–	–	–	–	–	0.26	No	No	–
6. Hair loss	–	–	0.33	–	–	0.31	No	No	–
7. Skin rash	–	–	0.47	–	–	0.45	Yes (F3)	Yes (F3)	F3
8. Red/painful eyes	–	0.41	–	–	0.37	0.53	No	No	–
9. Pain while breathing	0.75	–	–	–	–	0.59	Yes (F1)	Yes (F1)	F1
10. Shortness of breath	0.64	–	–	–	–	0.49	Yes (F1)	Yes (F1)	F1
11. 'White' fingers in cold weather	–	–	0.36	–	–	0.26	No	No	–
12. Itch	–	–	–	–	–	0.21	No	No	–
13. Ankle oedema	0.31	–	0.36	–	–	0.36	No	No	–
14. Chubby cheeks/face	–	–	0.45	–	–	0.53	Yes (F3)	Yes (F3)	F3
15. Increased appetite	–	–	–	0.73	–	0.72	Yes (F4)	Yes (F4)	F4
16. Decreased appetite	0.30	–	–	−0.69	–	0.56	No	Yes (F4)	–
17. Pimples/acne	–	0.37	–	–	−0.55	0.50	No	No	–
18. Facial hair growth	–	–	0.73	–	–	0.53	Yes (F3)	Yes (F3)	F3
19. Blue/purple stretch marks	–	–	0.74	–	–	0.52	Yes (F3)	Yes (F3)	F3
20. Spontaneous bruises	–	–	0.42	–	–	0.42	Yes (F3)	Yes (F3)	–
21. Poor wound healing	–	–	0.30	–	–	0.31	No	No	–
22. Muscle weakness	0.58	–	–	–	–	0.63	Yes (F1)	Yes (F1)	F1
23. Blurred vision	–	0.63	–	–	–	0.54	Yes (F2)	Yes (F2)	F2
24. Nightmares	–	0.58	–	–	–	0.35	Yes (F2)	Yes (F2)	F2
25. Mood changes	–	0.44	–	–	–	0.48	Yes (F2)	No	F2
26. Nausea/vomiting	–	0.46	–	–	–	0.45	Yes (F2)	No	F2
27. Stomach complaints	–	0.36	–	–	–	0.41	No	No	–
28. Sensitivity to sunlight	–	–	–	–	0.38	0.47	No	No	F5
29. Sensitivity to artificial light	–	0.30	–	–	0.35	0.41	No	No	F5
30. Fits/seizures	–	–	0.47	–	–	0.30	Yes (F3)	Yes (F3)	–
31. Fainting	–	0.59	–	–	–	0.48	Yes (F2)	Yes (F2)	F2
32. Genital sores	–	0.47	–	–	–	0.21	Yes (F2)	Yes (F2)	–
33. Chest pain	0.55	–	–	–	–	0.47	Yes (F1)	Yes (F1)	F1
34. Loss of concentration	–	0.79	–	–	–	0.70	Yes (F2)	Yes (F2)	F2
35. Muscle cramps	0.41	–	–	–	–	0.46	Yes (F1)	No	–
36. Vulnerable skin	–	–	0.55	–	–	0.52	Yes (F3)	Yes (F3)	–
37. Disturbed memory	–	0.78	–	–	–	0.58	Yes (F2)	Yes (F2)	F2
38. Weight gain	–	–	–	0.64	–	0.68	Yes (F4)	Yes (F4)	F4

Note. Loadings ≥ |0.30| shown; factors labelled by Tucker congruence with the full-sample solution (F1 pain/somatic-systemic; F2 neuropsychological/mood; F3 skin/treatment-related; F4 appetite/weight; F5 photosensitivity). Rule: primary loading ≥ 0.40 and no secondary loading ≥ 0.30; no post hoc deviations applied. All 23 short-form items kept their full-sample factor as primary factor; the photosensitivity items (28, 29) did not reach the 0.40 threshold, indicating that this two-item factor is the least stable component of the short form.
